# Endothelial Cells Promote Docetaxel Resistance of Prostate Cancer Cells by Inducing ERG Expression and Activating Akt/mTOR Signaling Pathway

**DOI:** 10.3389/fonc.2020.584505

**Published:** 2020-12-16

**Authors:** Wenhao Zhou, Yiming Su, Yu Zhang, Bangmin Han, Haitao Liu, Xiaohai Wang

**Affiliations:** Department of Urology, Shanghai General Hospital, Shanghai Jiao Tong University School of Medicine, Shanghai, China

**Keywords:** endothelial cells, docetaxel, chemoresistance, prostate cancer, FGF2

## Abstract

Docetaxel is a first-line chemotherapy for the treatment of patients with castration-resistant prostate cancer (CRPC). Despite the good initial response of docetaxel, drug resistance will inevitably occur. Mechanisms underlying docetaxel resistance are not well elaborated. Endothelial cells (ECs) have been implicated in the progression and metastasis of prostate cancer. However, little attention has been paid to the role of endothelial cells in the development of docetaxel resistance in prostate cancer. Here, we sought to investigate the function and mechanism of endothelial cells involving in the docetaxel resistance of prostate cancer. We found that endothelial cells significantly promoted the proliferation of prostate cancer cells and decreased their sensitivity to docetaxel. Mechanistically, basic fibroblast growth factor (FGF2) secreted by endothelial cells leads to the upregulation of ETS related gene (ERG) expression and activation of the Akt/mTOR signaling pathway in prostate cancer cells to promote docetaxel resistance. In summary, these findings demonstrate a microenvironment-dependent mechanism mediating chemoresistance of prostate cancer and suggest that targeting FGF/FGFR signaling might represent a promising therapeutic strategy to overcome docetaxel resistance.

## Introduction

Prostate cancer is one of the most common malignant tumors in the male genitourinary system and its mortality rate ranks second in male malignancies (1). For advanced prostate cancer, androgen deprivation therapy (ADT) is the standard of care. However, after 12 to 18 months of ADT, most patients become insensitive to it and gradually develop into castration-resistant prostate cancer (CRPC) (2). In 2004, the landmark study TAX327 demonstrated a significant survival benefit for docetaxel over mitoxantrone, which was the first study to lay the foundation for chemotherapy in CRPC patients. Docetaxel treatment achieved PSA decline, prolonged overall survival (OS), and improved quality of life (3). Recently, STAMPEDE and CHAARTED trials have confirmed that docetaxel combined with ADT as the front-line therapy for metastatic hormone-sensitive prostate cancer (mHSPC) accomplished a dramatic survival advantage over ADT alone (4, 5). These encouraging results further strengthen the position and application of docetaxel in advanced prostate cancer. However, chemoresistance remains a significant obstacle to docetaxel treatment, which significantly decreases its clinical efficacy (6). Mechanisms of docetaxel resistance are not completely understood. Known mechanisms of resistance to docetaxel include increased expression of multidrug resistance (MDR) genes, tubulin alterations, deregulation of growth factors, and intracellular signaling pathways activation, tumor microenvironment, etc (7–11). Identification of specific mechanisms that modulate resistance to docetaxel will facilitate the development of novel therapies and improve responses to currently available therapies.

Swarnali Acharyya et al. first described that endothelial cells were involved in the development of chemoresistance of breast cancer *via* secretion of TNF-α (12). Increasing evidence has demonstrated that endothelial cells contribute to chemoresistance of cancer cells by secreting soluble factors in a paracrine fashion in lymphoma (13–15), glioblastoma (16, 17), colorectal cancer (18, 19), and other cancer types (20–23). Results from these studies showed that secreted factors including exosomes from endothelial cells can activate “cancer-advancing” signaling pathways in cancer cells such as AKT, Wnt, NOTCH, and epithelial-mesenchymal transition pathways in favor of survival under chemotherapy. Our previous studies manifested that endothelial cells promoted the metastasis of prostate cancer by enhancing autophagy and increasing IL-6 secreion (24, 25), which indicated that endothelial cells are associated with prostate cancer progression. However, whether endothelial cells play a role in the development of docetaxel resistance in prostate cancer remains largely elusive.

Therefore, we set out to assess the effect of endothelial cells on the evolution of chemoresistance in prostate cancer. Herein, we demonstrated that endothelial cells significantly promoted prostate cancer cell proliferation and chemoresistance *via* constructing a co-culture system *in vitro*. Mechanistically, we showed that endothelial cells-derived FGF2 mediated ERG expression and Akt/mTOR activation in prostate cancer cells. Furthermore, we utilized a subcutaneous xenograft tumor model to verify the results of *in vitro* experiments *in vivo*. Overall, these findings demonstrated a synergistic role of endothelial cells in contributing to prostate cancer proliferation and chemoresistance *via* inducing ERG expression and activating the Akt/mTOR signaling pathway.

## Materials and Methods

### Cell Lines and Treatment

Human umbilical vein endothelial cells (HUVEC), 22Rv1, and C4-2B were purchased from the American Type Culture Collection (ATCC). HUVEC was cultured in Dulbecco’s Modified Eagle Medium supplemented with 10% fetal bovine serum (FBS; Gibco). 22Rv1 and C4-2B were cultured in RPMI 1640 medium with 10% fetal bovine serum (FBS; Gibco) and 1% penicillin-streptomycin. Cells were maintained at 37°C and 5% of CO2 in humidified air. Human FGF-basic(FGF2) (PeproTech, NJ, USA), Human IL-6(PeproTech, NJ, USA), Human IL-8(PeproTech, NJ, USA), Human RANTES (PeproTech, NJ, USA), PD173074 (Selleckchem, TX, USA), and Perifosine (Selleckchem, TX, USA) were used for this study.

### Co-Culture Experiments

Co-culture experiments were performed by seeding prostate cancer cells in the lower chamber and HUVEC in the upper chamber of a 6-well or a 24-well transwell apparatus with a 0.4 um pore size (Corning, NY, USA). The medium used in the co-culture model was composed of 1640 and DMEM (with a ratio 1:1) supplemented with 10% FBS (e.g., In a 6-well transwell apparatus, 1.5 ml 1640 supplemented with 10% FBS was added to the lower chamber and 1.5 ml DMEM supplemented with 10% FBS to the upper chamber. In a 24-well transwell apparatus, 0.5ml 1640 supplemented with 10% FBS was added to the lower chamber and 0.5ml DMEM supplemented with 10% FBS to the upper chamber.)

### Proliferation Assay

22Rv1 and C4-2B cell proliferation was determined using a Cell Counting Kit (Dojindo, Kumamoto, Japan) and EdU DNA Cell Proliferation Kit (RiboBio, Guangzhou, China) without trypsinization. For the CCK-8 assay, 2 × 10^3^ 22Rv1 or C4-2B cells for each well were seeded in 24-well plates alone or in the lower chamber of a 24-well transwell apparatus co-cultured with 2 × 10^3^ HUVEC for each well. At various time points, 30 μl CCK-8 solution with 300 μl PBS was added to each well and incubated at 37°C for 2 h. The absorbance at 450 nm was measured with a microplate reader (Bio-Rad Laboratories) and marked as OD450. Similarily, 2 × 10^3^ 22Rv1 or C4-2B cells for each well were seeded in 24-well plates alone or in the lower chamber of a 24-well transwell apparatus co-cultured with 2 × 10^3^ HUVEC for each well in triplicate. EdU experiments were carried out for analyzing the proliferation of 22Rv1 and C4-2B cells after -48h according to the manufacturer’s instructions. The percentage of EdU positive cells (EdU positive/DAPI positive) was labeled as EdU positive cells (%). Three technical replicates were done per experiment, and three independent experiments were performed.

#### Cell Viability Assay

22Rv1 and C4-2B cell viability was determined using a Cell Counting Kit (Dojindo, Kumamoto, Japan) without trypsinization. 1x10^4^ 22Rv1 or C4-2B cells were cultured with or without 1x10^4^ HUVEC in 24-well plates overnight at 37°C. After 24h, the prostate cancer cells were then treated with docetaxel at indicated concentrations for 48h. The number of survival cells was detected at the absorbance of 450 nm by a microplate reader (Bio-Rad Laboratories). Three technical replicates were done per experiment, and three independent experiments were performed. IC50 was calculated using nonlinear regression after logarithms transformation by Graphpad Prism 7.0 software.

### Apoptosis Analysis

Cells were dissociated by 0.25% trypsin-EDTA and harvested by centrifugation, rinsed once with PBS, and suspended in binding buffer. Annexin V and PI staining were performed as per the manufacturer’s instructions (BD Biosciences). After incubation, cells were measured by Beckman Coulter Accuri Cytometers C6 flow cytometer (BD) and analyzed using the Accuri CFlow software. Three technical replicates were done per experiment, and three independent experiments were performed. The apoptotic rate is based on the flow cytometry analysis of cells labelled with FITC‐Annexin V and PI which allows detection of cells in early and late stages of apoptosis.

### Quantitative Real-Time PCR

Total RNA of HUVEC, 22Rv1, and C4-2B was extracted using TRIzol (Life Technologies, Carlsbad, CA). Reverse transcript PCR for mRNA was carried out using Prime Script RT Master Mix (Takara, Otsu, Shiga, Japan) according to the manufacturer’s instructions. Real-time PCR was performed using SYBR Premix Ex Taq (TaKaRa, Japan) according to the manufacturer’s instructions. Primers used were: ERG sense, 5’CGCAGAGTTATCGTGCCAGCAGAT3’; antisense, 5’CCATATTCTTTCACCGCCCACTCC3’; GAPDH sense, 5’AATGTCACCGTTGTCCAGTTG3’; antisense, 5’ GTGGCTGGGGCTCTACTTC3’; FGF2 sense, 5’ AGAAGAGCGACCCTCACATCA3’; antisense, 5’ CGGTTAGCACACACTCCTTTG3’; FGFR sense, 5’ GTGATGAAGATAGCAGACTTTGG3’; antisense, 5’ GAGCCCCCCAGAGTGAAGATCTC3’; Expression levels were normalized to the expression of GAPDH RNA. Three technical replicates were done per experiment, and three independent experiments were performed.

### Western Blot

Total proteins were extracted from tissues and primary cells in lysis buffer on ice. The lysates were cleared by centrifugation at 12,000 g at 4°C for 15 min, and the protein concentrations were measured by BCA protein assay kit. Protein extracts were heated at 95°C with 5 × SDS-PAGE Loading Buffer (Dingguo, WB-0091) for 10 min and separated by SDS-polyacrylamide gel electrophoresis and transferred to a polyvinylidene fluoride membrane. After blocking with 5% skim milk, primary antibodies against ERG(Cell Signaling Technology, #97249, 1:1,000 dilution), AR(Cell Signaling Technology, #3202, 1:1,000 dilution), Caspase3(Cell Signaling Technology, #9662, 1:1,000 dilution), PARP(Cell Signaling Technology, #9532, 1:1,000 dilution), Akt(Cell Signaling Technology, #9272, 1:1,000 dilution), phospho-Akt (Ser473)(Cell Signaling Technology, #4060, 1:1,000 dilution), mTOR (Cell Signaling Technology, #2972, 1:1,000 dilution), phospho-mTOR(Cell Signaling Technology, #2971, 1:1,000 dilution), GAPDH(Sangon #D110016, 1:3,000 dilution) were used at 4°C overnight. After being washed three times with TBS-T, the blots were incubated with horseradish peroxidase-conjugated secondary antibodies for 1 h and visualized by enhanced chemiluminescence assay (ECL, Thermo). Three technical replicates were done per experiment, and three independent experiments were performed. Western blots were relatively quantified as a ratio of each protein band relative to the lane’s loading control by measuring mean gray value with Image J.

### Lentivirus Construction and Infection

The coding sequence of ERG mRNA (NM_001136154.1) was synthesized and cloned into pLenti6.3 lentivirus overexpression vectors. All lentiviruses were constructed by Shanghai Obio Technology Company. After packaging, pLenti6.3-ERG lentivirus and negative control lentivirus were used to infect C4-2B cells, for the construction of ERG overexpressing C4-2B (C4-2B-ERG) and negative control C4-2B cells. Standard biosecurity and institutional safety procedures were strictly observed.

### RNA Interference

The siRNA targeting ERG mRNA or negative control siRNAs were designed and constructed by GenePharma Company. ERG siRNA sequences are as follows: siERG1: sense 5’CCACGGUUAAUGCAUGCUATT 3’, antisense 5’ UAGCAUGCAUUAACCGUGGAG3’. siERG2: sense5’ GCUAUGGAGUACAGACCAUTT 3’, anti-sense: 5’ AUGGUCUGUACUCCAUAGCTT3’. C4-2B-ERG cells were transfected with ERG siRNAs and negative control siRNAs according to the manufacturer’s protocol.

### Cytokine Antibody Arrays and ELISA assay

Human Cytokine Antibody Arrays G-Series 1000 (Raybiotech) was used according to the manufacturer’s instructions. Briefly, serum-free media from prostate cancer cells culture and HUVEC/prostate cancer cells co-culture were collected and incubated with the blocked glass chips for 2 h at room temperature. After development, the signals (532 nm excitation) were scanned and extracted using the InnoScan 300 Microarray Scanner (Innopsys, Inc. France). The results were analyzed using the RayBiotech Q Analyzer program.

HUVEC cells were cultured in DMEM with 10% fetal bovine serum until 80% of confluency. These cells were then washed with PBS and cultured with or without prostate cancer cells in fresh serum-free media. Cell culture media were collected after 24 h. FGF2 level was determined by using a commercial human FGF2 ELISA kit (RayBiotech, GA, USA) according to the manufacturer’s instructions.

### Immunohistochemical Staining

Paraffin sections were dewaxed in xylene and rehydrated in graded ethanol, followed by incubation with non-specific protein blocking solution 1% bovine serum albumin in PBS for 45 min at room temperature, and incubated with primary antibody against ki67 (Cell Signaling Technology, #9449, 1:400) overnight at 4°C. The sections were then incubated with HRP‐conjugated secondary antibody for 60 min at room temperature followed by treatment with diaminobenzidine (Sigma-Aldrich) working solution and counterstaining with hematoxylin. Image-Pro Plus software was applied for the quantitative analysis of immunohistochemical results. Three immunohistochemical images of each group were taken to measure the average optical density.

### TUNEL Assay

The sections were dewaxed with xylene twice for 15 min and then treated with a graded series of alcohol (100%, 95%, 85%, 70%, and 50% ethanol in double-distilled H_2_O) and rehydrated in PBS (pH 7.5). Apoptotic cells were detected according to the protocol of the TUNEL kit (Roche, Mannheim, Germany). DAPI was then used for counterstaining of the nuclei and images were obtained by laser scanning microscopy. Three technical replicates were done per experiment, and three independent experiments were performed. The apoptosis index was based on the fluorescence microscopy detection of positive TUNEL cells and positive DAPI cells.

### Animal Experiment

Ten male BALB/c nude mice (five to six-week-old) were purchased from Animal Center of the Chinese Academy of Sciences (Shanghai, China) and all animal studies were carried out in compliance with guidelines of the Chinese Council on Animal Care. Protocols were approved by the Medical Science Ethics Committee of Shanghai General Hospital. Five mice were treated with 22Rv1 cells alone, the other five mice were treated with 22Rv1 cells plus HUVEC. Specially, 5×10^6^ 22Rv1 cells mixed with or without 2.5×10^6^ HUVEC were suspended in 200 μl PBS diluted Matrigel (100 μl PBS+100μl Matrigel), which were subcutaneously inoculated into the right flanks of mice. When tumors reached a mean size of 150 mm^3^, the mice were treated with docetaxel 20 mg/kg intraperitoneally once a week for four weeks. Every six days, the length (L) and width (W) of tumors were measured using calipers, and their volumes were calculated using the equation (L × W^2^/2).

### Statistical Analysis

The data were expressed as mean ± SD and the two groups were compared with the independent samples *t*-test. Statistical analysis was performed by using the SPSS software package (version19.0) and Graphpad Prism 7.0. A *P* value < 0.05 indicated a significant difference.

## Results

### Endothelial Cells Promote the Proliferation of Prostate Cancer Cells With or Without Docetaxel Treatment

**Figure 1:**
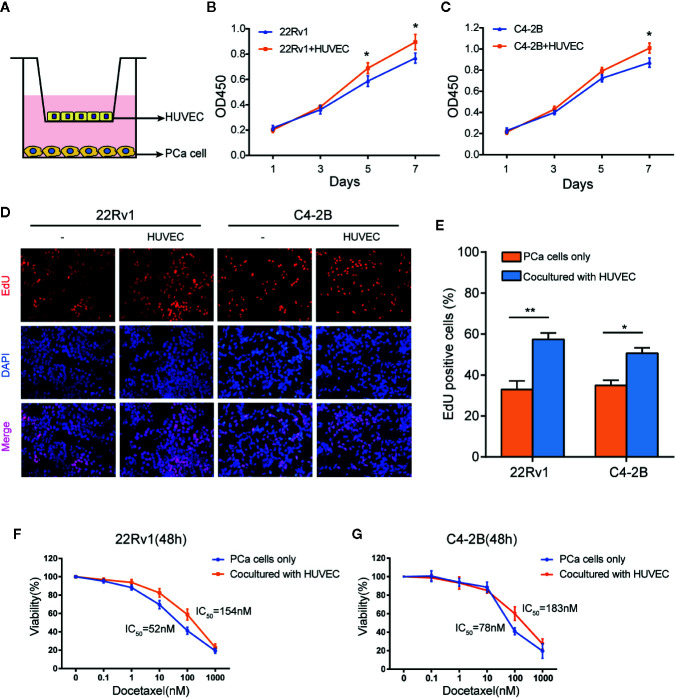
Endothelial cells enhance the proliferation of prostate cancer cells with or without docetaxel treatment. **(A)** This picture showed the construction of the co-culture system by using a 0.4 μm pore Transwell plate. **(B**, **C)** CCK8 assays revealed that the co-culture group with human umbilical vein endothelial cells (HUVEC) increased the growth rate of 22Rv1 and C4–2B compared with the respective monoculture group. **(D**, **E)** EdU assays demonstrated proliferation differences between 22Rv1 cultured alone and 22Rv1 co-cultured with HUVEC, C4–2B cultured alone, and C4–2B co-cultured with HUVEC. **(F, G)** Cell viability of 22Rv1 and C4–2B (cultured with or without HUVEC) treated with docetaxel at the indicated concentrations for 48 h. The data represent the mean ± SD and differences were tested by the Student’s t-test; *P < 0.05; **P < 0.01.

We established a co-culture system (media as control) to investigate the effect of endothelial cells on the growth of prostate cancer cells (**Figure 1A**). 22Rv1 and C4-2B cells were cultured with or without HUVEC. CCK8 assays revealed that 22Rv1 and C4-2B cells co-cultured with HUVEC increased cell proliferation capability compared with the corresponding monoculture group, respectively (**Figures 1B, C**). Moreover, as shown in **Figures 1D, E**, 22Rv1 or C4-2B cells co-cultured with HUVEC incorporated more EdU than the monoculture group (58% *vs* 32% for 22Rv1 and 52% *vs* 34% for C4-2B, respectively). To investigate whether HUVEC has a synergistic impact on docetaxel resistance in prostate cancer cells, we measured cell viability of 22Rv1 and C4–2B cultured with or without HUVEC after 48h of docetaxel treatment at the indicated concentration. Interestingly, 22Rv1 and C4–2B co-cultured with HUVEC demonstrated lower sensitivity to docetaxel than the monoculture group (**Figures 1F, G**). Collectively, these results indicated that endothelial cells may enhance the proliferation of prostate cancer cells before and after docetaxel treatment.

### Endothelial Cells Inhibit the Apoptosis of Prostate Cancer Cells After Docetaxel Treatment

**Figure 2:**
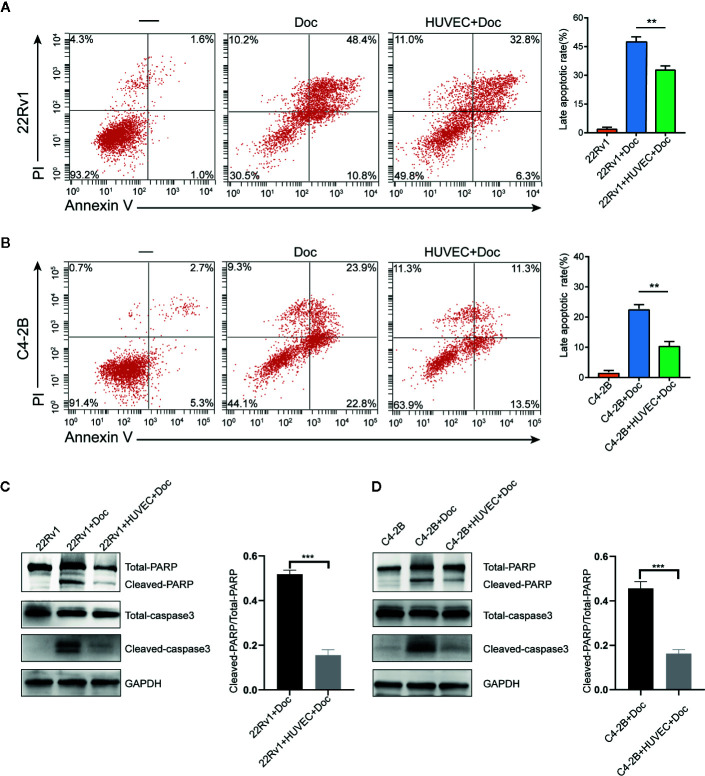
Endothelial cells suppress the apoptosis of prostate cancer cells after docetaxel treatment. **(A, B)** The percentage of apoptosis of 22Rv1 and C4–2B cells cultured with or without human umbilical vein endothelial cells (HUVEC) after 10 nM docetaxel treatment for 48 h. **(C, D)** Expression of different apoptosis-related proteins in 22Rv1 and C4–2B cultured with or without HUVEC was detected by western blot. Data were presented as mean ± SD; **P < 0.01, ***P < 0.001.

We performed flow cytometry to calculate the apoptosis rates of 22Rv1 and C4–2B cultured with or without HUVEC after treatment with 10 nM docetaxel for 48 h. Surprisingly, the apoptosis rates of 22Rv1 and C4–2B in the co-culture group decreased significantly compared with the monoculture group, which suggested that HUVEC could shield prostate cancer cells from docetaxel-induced apoptosis (**Figures 2A, B**). Moreover, western blotting assay showed that the expression of apoptosis proteins including cleaved-PARP and cleaved caspase-3 was down-regulated markedly in the co-culture group treated with docetaxel compared with the monoculture group treated with docetaxel (**Figures 2C, D**). Taken together, these data manifested that endothelial cells can inhibit the apoptosis of prostate cancer cells after docetaxel treatment.

### ERG Is a Key Mediator for Endothelial Cell-Induced Docetaxel Resistance in Prostate Cancer Cells

**Figure 3:**
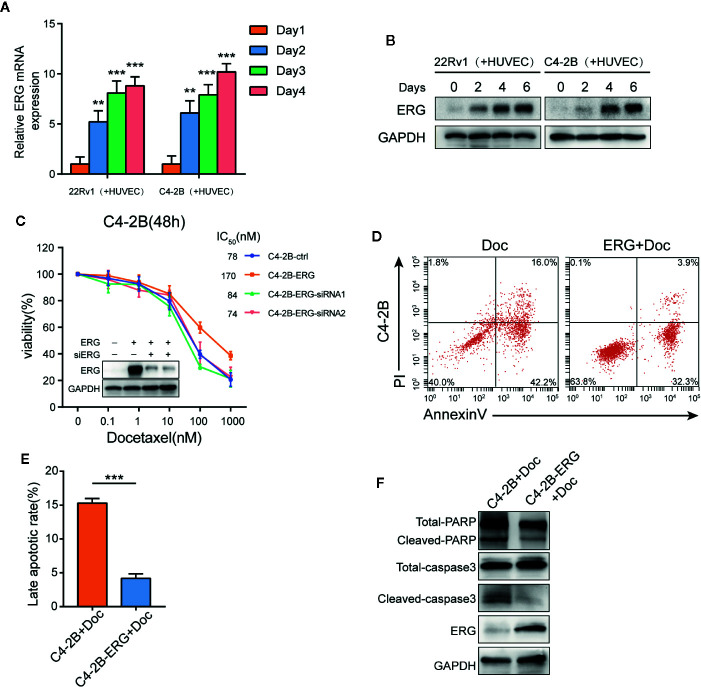
ETS related gene (ERG) is a key mediator for endothelial cells-induced docetaxel resistance in prostate cancer cells. **(A, B)** 22Rv1 and C4–2B cells were co-cultured with human umbilical vein endothelial cells (HUVEC) for the indicated days, qPCR and western blot analysis were performed to assess ERG expression. **(C)** Cell viability of C4–2B cells transfected with ERG and siRNA targeting ERG incubated with the indicated dose of docetaxel for 48h. Western blot was conducted to measure ERG expression. **(D, E)** The percentage of apoptotic C4–2B and C4-2B-ERG cells treated with 10 nM docetaxel for 48 h. **(F)** The expression of total/cleaved PARP and caspase-3 in C4–2B and C4-2B-ERG cells treated with docetaxel was determined by western blotting. Data were presented as mean ± SD; **P < 0.01; ***P < 0.001.

Recent studies have revealed that ERG can promote resistance to docetaxel in different prostate cancer cell lines (26, 27). Accordingly, the expression of ERG in 22Rv1 and C4–2B cells co-cultured with HUVEC was determined, respectively. Interestingly, qPCR and western blot assay demonstrated that the expression of ERG increased in prostate cancer cells with prolonged co-culture time (**Figures 3A, B**). This finding indicated that endothelial cells could induce ERG expression in prostate cancer cells in an AR independent manner. To investigate whether ERG expression has an impact on docetaxel resistance, we constructed ERG overexpressing C4-2B cells (C4-2B-ERG) by lentiviral transfection. Cell viability assays revealed that ERG overexpression enabled more C4-2B cells to survive upon the same dose of docetaxel while knocking down ERG could restore their sensitivity to docetaxel (**Figure 3C**). Annexin V/PI assay was used to assess apoptosis following docetaxel treatment in C4-2B with or without ERG overexpression. The percentage of apoptotic cells was much lower in C4-2B-ERG cells than that in C4-2B cells (**Figures 3D, E**). Consistently, cleaved-caspase3 and cleaved-PARP also decreased in C4-2B-ERG cells compared with C4-2B cells by western blot analysis (**Figure 3F**). Collectively, these data suggested that endothelial cells enhance the docetaxel resistance of prostate cancer cells through increasing ERG expression.

### FGF2 Secreted From Endothelial Cells to PCa/ECs Co-Culture System Induces ERG Expression and Docetaxel Resistance in Prostate Cancer Cells

**Figure 4:**
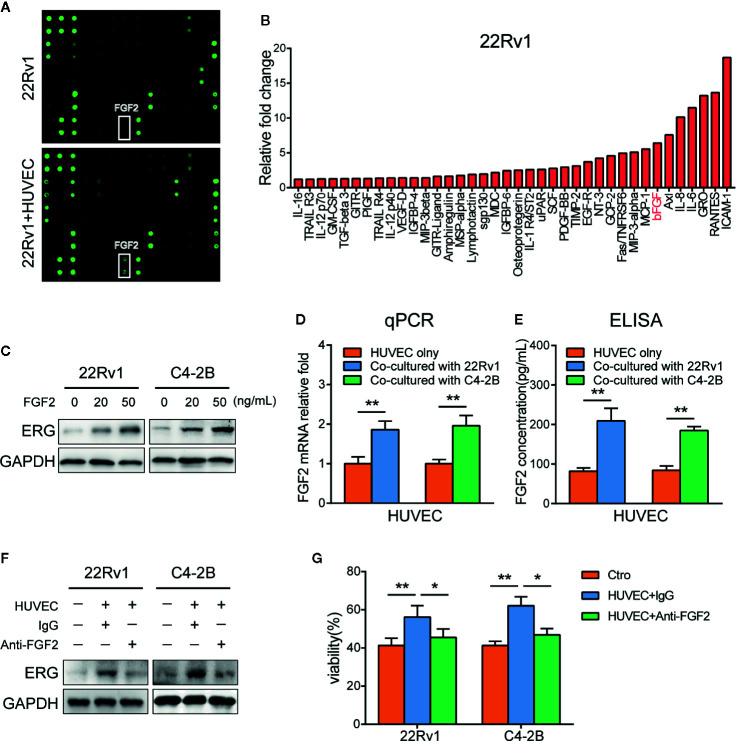
FGF2 secreted from endothelial cells induces ETS related gene (ERG) expression and docetaxel resistance in prostate cancer cells. **(A)** The cytokines array analysis. The conditioned media collected from 22Rv1, 22Rv1, and human umbilical vein endothelial cells (HUVEC) co-culture systems were used for the analysis. **(B)** Histogram showing increased cytokines in 22Rv1 and HUVEC co-culture supernatant compared to 22Rv1 culture supernatant. **(C)** Western blot analysis of ERG expression following FGF2 treatment in prostate cancer cells. 22Rv1 and C4–2B cells (1×10^5^/well) were treated with 20 ng/ml and 50 ng/ml of FGF2 for 48 h, respectively. **(D)** Quantitative PCR analysis of FGF2 expression in HUVEC cultured with or without prostate cancer cells. **(E)** ELISA demonstrating different FGF2 concentrations in culture supernatants from HUVEC and HUVEC co-cultured with prostate cancer cells. **(F, G)** 22Rv1 and C4-2B cells were cultured with or without HUVEC in the presence or absence of anti-FGF2 for 48h. **(F)** Western blot analysis of ERG expression of prostate cancer cells was conducted. **(G)** Cell viability assay of prostate cancer cells treated with 10 nM docetaxel for 48 h was performed. Data were presented as mean ± SD; *P < 0.05; **P < 0.01.

To determine which factor contributes to the expression of ERG in prostate cancer cells co-cultured with HUVEC, we conducted cytokines array according to the manufacturer’s protocol. As shown in **Figures 4A, B**, a series of cytokines were elevated in the culture media of 22Rv1 co-cultured with HUVEC compared with 22Rv1 cultured alone. Among these factors, IL-6, IL-8, RANTES(CCL5), and bFGF (FGF2) have been identified as cytokines that are associated with chemotherapy resistance (28–31). Next, we examined the effect of IL-6, IL-8, CCL5, and FGF2 in the regulation of ERG expression by adding them to the culture media of prostate cancer cells, respectively. As demonstrated in **Figure 4C**, FGF2 upregulated the expression of ERG in 22Rv1 and C4–2B cells by western blotting analysis, while IL-6, IL-8, and CCL5 did not (data not shown). Interestingly, the expression of FGF2 increased in HUVEC co-cultured with prostate cancer cells compared with HUVEC cultured alone measured by qPCR and ELISA analysis (**Figures 4D, E**). Moreover, blocking FGF2 by using PD173074 could reverse the enhancing effects of HUVEC on ERG expression and docetaxel resistance in prostate cancer cells (**Figures 4F, G**).

Furthermore, to investigate whether there was a switch to an autocrine expression by prostate cancer cells in our co-culture model, we performed additional assays of FGF2 expression in 22Rv1 and C4-2B cells in the monoculture and co-culture system. We compared it with that in HUVEC cells. Interestingly, the expression of FGF2 in 22Rv1 and C4-2B cells co-cultured with HUVEC cells did not change within 48 h compared with that in 22Rv1 and C4-2B cells cultured alone measured by qPCR (**Figure S1A, B**). Moreover, the expression of FGF2 in 22Rv1 and C4-2B cells co-cultured with HUVEC for 48 h was relatively low compared with that in HUVEC cells in the co-culture system measured by qPCR (**Figure S1C, D**).

In addition, to examine whether FGF2 expression was regulated by docetaxel, we carried out additional assays of FGF2 expression in HUVEC co-cultured with prostate cancer cells following docetaxel treatment. Interestingly, the expression of FGF2 in HUVEC co-cultured with prostate cancer cells following docetaxel treatment (10nM for 48h) did not change compared with that in HUVEC co-cultured with prostate cancer cells without docetaxel treatment measured by qPCR (**Figure S2A, S2B**). Moreover, the expression of FGF2 in 22Rv1 and C4-2B cells co-cultured with HUVEC cells following docetaxel treatment did not change compared with that in 22Rv1 and C4-2B cells co-cultured with HUVEC cells without docetaxel treatment (**Figure S2C, D**).

Besides, to ascertain whether FGF-R expression is affected by HUVEC or docetaxel in prostate cancer cells, we conducted additional assays of FGF-R expression in 22Rv1 and C4-2B cells cultured alone or co-cultured with HUVEC with or without docetaxel treatment. Interestingly, the expression of FGFR in 22Rv1 and C4-2B cells co-cultured with HUVEC cells did not change within 48h compared with that in 22Rv1 and C4-2B cells cultured alone measured by qPCR (**Figure S3A, B**). Moreover, FGF-R expression in 22Rv1 and C4-2B cells co-cultured with HUVEC cells following docetaxel treatment did not change compared with that in 22Rv1 and C4-2B cells co-cultured with HUVEC cells without docetaxel treatment (**Figure S3C, D**).

Taken together, these results revealed that FGF2 derived from HUVEC might represent the primary source of FGF2 in the co-culture system and play a key role in inducing the expression of ERG and promoting chemoresistance to docetaxel in prostate cancer cells. The indicated docetaxel treatment did not cause the expression of FGF2 in HUVEC and prostate cancer cells in the co-culture system. Co-culture with HUVEC or docetaxel treatment did not promote the production of FGFR in prostate cancer cells within the indicated culture period and concentration range.

### Endothelial Cells Induced Docetaxel Resistance *via* FGF2/ERG/Akt/mTOR Signaling Pathway in Prostate Cancer Cells

**Figure 5:**
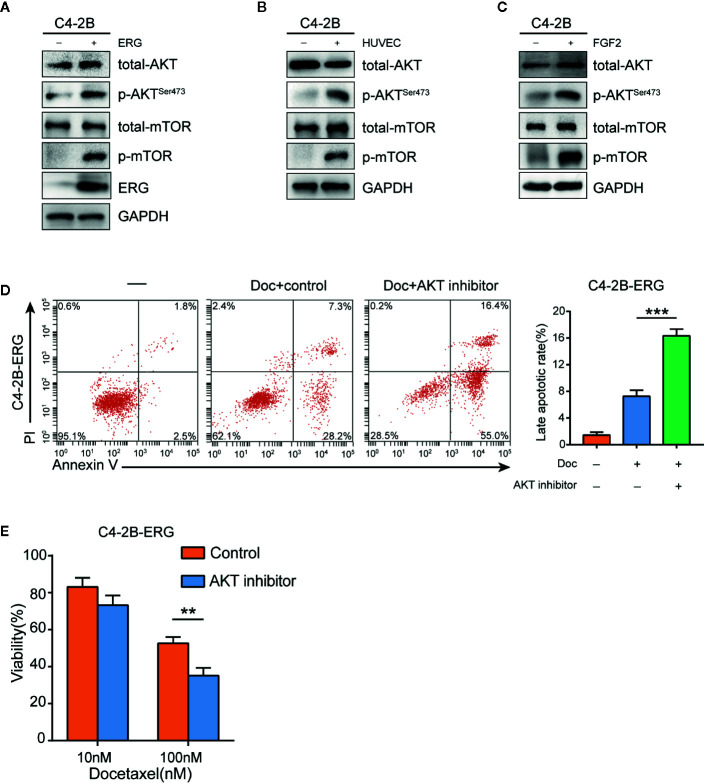
ETS related gene (ERG) is involved in the regulation of the Akt/mTOR signaling pathway in prostate cancer cells. **(A)** Western blotting analysis of total Akt, phosphorylated Akt (p-Akt), total mTOR, phosphorylated mTOR (p-mTOR) proteins in C4–2B and C4-2B-ERG cells. **(B)** C4–2B cells were co-cultured with human umbilical vein endothelial cells (HUVEC) for 48 h, and total Akt, phosphorylated Akt (p-Akt), total mTOR, phosphorylated mTOR (p-mTOR) proteins were measured subsequently by western blotting. **(C)** C4–2B cells were treated with 50 ng/ml FGF2 for 48 h, western blotting analysis was performed subsequently for total Akt, phosphorylated Akt (p-Akt), total mTOR, phosphorylated mTOR (p-mTOR) proteins. **(D)** The apoptosis rates of C4-2B-ERG cells were determined by flow cytometry following docetaxel treatment with or without Akt inhibitor. **(E)** Cell viability of C4-2B-ERG cells treated with docetaxel at the indicated doses with or without Akt inhibitor for 48h. Data were presented as mean ± SD; **P < 0.01; ***P < 0.001.

Previous studies have demonstrated that the Akt/mTOR signaling pathway plays an important role in docetaxel‐resistant CRPC (32). Therefore, we hypothesized that this pathway might be involved in ERG-mediating chemoresistance to docetaxel in prostate cancer cells. As expected, phosphorylation of Akt and mTOR increased in ERG-overexpressing C4-2B cells compared with normal C4-2B cells by western blot analysis, which indicated that ERG was involved in the activation of Akt/mTOR signaling pathway (**Figure 5A**). We subsequently examined the phosphorylation status of Akt and mTOR in C4-2B cells co-cultured with HUVEC and found that HUVEC induced the phosphorylation of Akt and mTOR in C4-2B cells (**Figure 5B**). Meanwhile, FGF2 treatment also upregulated the phosphorylation of Akt and mTOR in C4-2B cells (**Figure 5C**). To further investigate the key role of Akt/mTOR in chemoresistance mediated by ERG, we used Perifosine (an Akt inhibitor) in ERG-overexpressing C4-2B cells. Flow cytometry analysis indicated that the percentage of apoptotic C4-2B-ERG cells increased when they were treated with docetaxel plus Perifosine compared to docetaxel alone (**Figure 5D**). Furthermore, cell viability assay showed that Perifosine could decrease docetaxel resistance induced by ERG (**Figure 5E**). Collectively, these findings suggested that ERG enhances docetaxel resistance *via* the Akt/mTOR signaling pathway in prostate cancer cells.

### Endothelial Cells Promote the Docetaxel Resistance of Prostate Cancer Cells *In Vivo*

**Figure 6:**
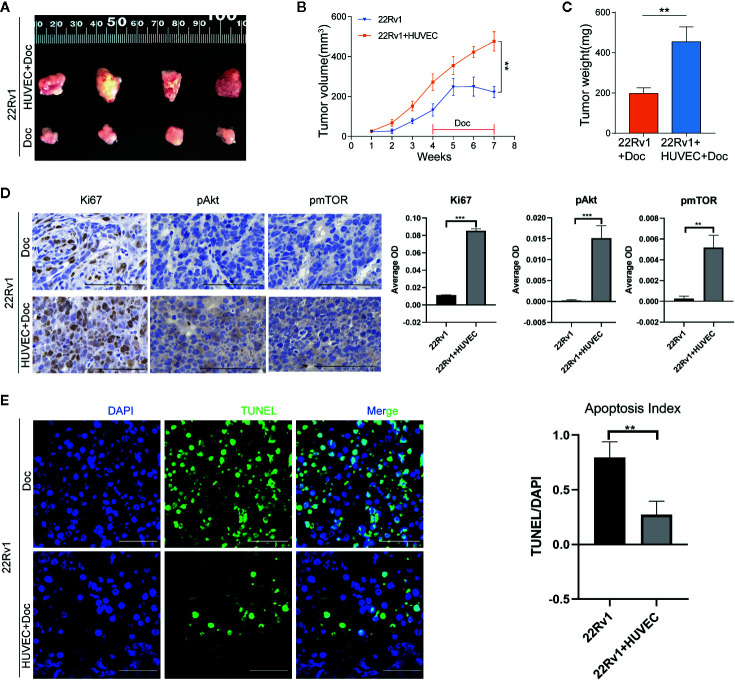
Endothelial cells promote the docetaxel resistance of prostate cancer cells *in vivo*. **(A–C)** 22Rv1 cells and 22Rv1 cells combined with human umbilical vein endothelial cells (HUVEC) were subcutaneously injected into the right flanks of athymic BALB/c nude mice, respectively. Mice were administered with docetaxel by intraperitoneal injection once a week for four weeks. Tumor volumes were calculated and tumor weight was measured. **(D)** Immunohistochemistry staining of Ki67, pAkt, pmTOR in excised xenograft tumors. Scale bars, 50 μm. **(E)** Representative fluorescent images of TUNEL-positive apoptotic tumor cells. Scale bars, 50 μm. Data were presented as mean ± SD; **P < 0.01.

To further validate the above findings *in vivo*, 22Rv1 cells with or without HUVEC were injected subcutaneously into the right flanks of athymic BALB/c nude mice. As shown in **Figures 6A–C**, co-injection of 22Rv1 cells with HUVEC dramatically enhanced tumor growth rate and sustained tumor proliferation in mice under docetaxel chemotherapy in comparison with 22Rv1 uni-injection group. Immunohistochemistry analysis revealed that the expression of Ki67, pAkt, pmTOR increased markedly in tumors derived from 22Rv1 co-injected with HUVEC compared to 22Rv1 injected alone following chemotherapy with docetaxel (**Figure 6D**). Besides, the TUNEL assay demonstrated that the percentage of TUNEL positive cells in tumors from the co-injection group was lower than that from the uni-injection group following docetaxel chemotherapy (**Figure 6E**). Collectively, these data suggested that endothelial cells not only promote prostate cancer cells proliferation but also shield prostate cancer cells from docetaxel-induced apoptosis *in vivo*.

### Summary of the Mechanism Underlying Endothelial Cells Promoting Docetaxel Resistance in Prostate Cancer Cells

Endothelial cells co-cultured with prostate cancer cells can secrete FGF2 into the culture media. FGF2 boosts the expression of the ERG gene in prostate cancer cells subsequently. ERG protein activates Akt/mTOR signaling pathway contributing to docetaxel resistance in prostate cancer cells ultimately.

## Discussion

**Figure 7:**
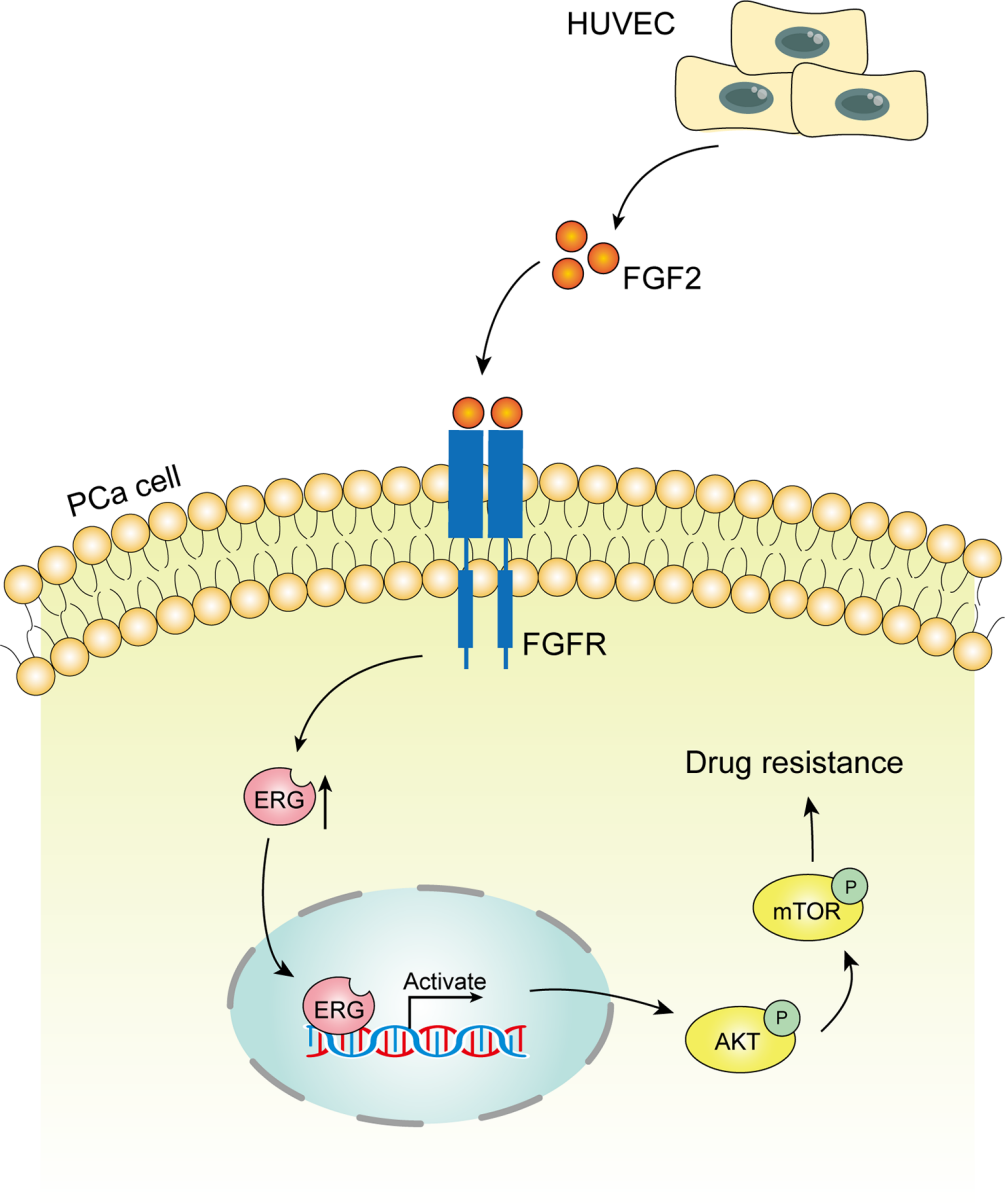
Schematic model of the hypothesized mechanism by which endothelial cells promote docetaxel resistance of prostate cancer cells. Endothelial cells co-cultured with prostate cancer cells can secrete FGF2 into the culture media. FGF2 boosts the expression of the ETS related gene (ERG) gene in prostate cancer cells subsequently. ERG protein activates Akt/mTOR signaling pathway contributing to docetaxel resistance in prostate cancer cells ultimately.

The tumor microenvironment is recognized to profoundly influence cellular response to chemotherapy. Endothelial cells are key components of the tumor microenvironment. Endothelial cells can secrete a variety of cytokines, which play important roles in tumor progression and metastasis (33). Recent studies have demonstrated that endothelial cells can adjust their response to chemotherapy. Meng F et al. (34) reported that endothelial cells from mouse liver cancer enhance their survival and migration in response to chemotherapeutic stress *via* an NF-κB-Akt-dependent manner. AKIYAMA K et al. (35) described that endothelial cells from melanoma acquire drug resistance to paclitaxel by multidrug resistance 1 (MDR1) upregulation *via* VEGF signaling. However, to the best of our knowledge, few studies have focused on the role of endothelial cells in modulating chemoresistance of cancer cells to docetaxel (17, 20). Therefore, appreciating the important experimental evidence of endothelial cells’ involvement in the chemoresistance of prostate cancer can have important therapeutic implications. In this study, we have identified endothelial cells as a mediator contributing to docetaxel resistance of prostate cancer cells for the first time. Endothelial cells secrete FGF2 in the tumor microenvironment to enhance the expression of ERG in prostate cancer cells. ERG protein stimulates the phosphorylation of Akt and mTOR subsequently. The activation of the Akt/mTOR signaling pathway promotes the docetaxel resistance of prostate cancer cells ultimately (**Figure 7**). Our results confirmed that endothelial cells contribute to the chemoresistance of prostate cancer cells in a paracrine manner. This new finding will shed valuable insights into the role of endothelial cells in the development of chemoresistance of prostate cancer cells and highlight the importance of endothelial cells as a therapeutic target in prostate cancer treatment.

The interaction of endothelial cells with tumor cells has been shown to mediate AR-independent invasion of prostate cancer cells (24). By analogy, we hypothesized that the communication between endothelial cells and prostate cancer cells may also play a role in modulating chemoresistance of prostate cancer. Consistently, we found that endothelial cells can enhance the chemoresistance of prostate cancer cells. Mechanistically, endothelial cells induce ERG expression in prostate cancer cells. ERG has been demonstrated to affect several parameters of microtubule dynamics and inhibit effective drug-target engagement of docetaxel with tubulin (26). Interestingly, we have discovered a novel way that induces ERG expression in prostate cancer. Through a combination of cytokines array, quantitative PCR, and ELISA assay, we confirmed that FGF2 was the key factor that facilitated the development of chemoresistance of prostate cancer cells co-cultured with endothelial cells. Gan, Wientjes (36) demonstrated that the expression of basic fibroblast growth factor correlates with resistance to paclitaxel in human tumors including prostate cancer. However, they did not further explore the underlying mechanism. Here, we identified a new signaling pathway that connects FGF2 and docetaxel resistance of prostate cancer. We showed that endothelial cells-derived FGF2 can induce ERG expression in prostate cancer cells in an AR-independent manner. This finding provides new evidence to the role of FGF2 in promoting chemoresistance of prostate cancer. However, when FGF2 antagonists were used to block the interactions between endothelial cells and prostate cancer cells, the expression of ERG could not be completely reversed. This may be due to there are other factors that exist in the tumor microenvironment to modulate the expression of ERG. Nevertheless, there are few reports focused on the regulation of ERG expression by cytokines. Kao C et al. (37) suggested that ERG expression could be modulated by EGF. Other AR-independent ways regulating ERG expression are needed to be further investigated in the future. Similarly, the enhancing effects of HUVEC on docetaxel resistance could not be completely reversed as well when FGF2 antagonists were added. This indicates that although FGF2 plays a significant role in mediating chemoresistance, there are other factors needed to be further investigated as well. Intriguingly, the expression of FGF2 in endothelial cells was elevated when co-cultured with prostate cancer cells compared with endothelial cells cultured alone. This finding implied that prostate cancer cells can give instructions to endothelial cells to adjust responses to external circumstances in favor of survival. The reciprocal crosstalk between tumor and microenvironment therefore substantially affects tumor cell biology. Although the ways and mechanisms by which prostate cancer cells act on endothelial cells are not the subject of this study. This reflection points out a new direction that needs further study in the future.

Moreover, the possibility of an additional autocrine mechanism deriving from prostate cancer cells merits further consideration. In the co-culture system, the diffusion of growth factors and signal molecules occurs bi-directionally, and it may be possible that HUVEC precondition prostate cancer cells to produce FGF2. It is likely that initially, FGF2 is expressed as a paracrine factor by stromal cells, and during cancer progression or following therapy, there is a switch to an autocrine expression. However, whether prostate cancer cells can secrete FGF2 autonomously is a controversial topic. Different studies have given different even conflicting answers. Cronauer et al. (38) demonstrated that DU-145 and PC-3 tumor cells produced FGF2 by ELISA and RT-PCR and prostate cancer cells was FGF2 positive by immunohistochemical staining. Nevertheless, Chandler et al. (39) documented that FGF2 mRNA was not detected in either PC‐3 or DU‐145 cell lines by RNase protection analysis. This finding was in contrast with the results of Cronauer (38) and Nakamoto et al (40) who detected FGF2 mRNA in both PC‐3 and DU‐145 cells by Northern analysis of poly(A) mRNA. The reasons for these conflicting results were unclear. Various assays and cell sources may explain the difference.

Furthermore, some studies found that the expression of FGF2 was detected in tumor stroma rather than tumor parenchyma. Sinowatz et al  (41) reported that immunostaining of FGF2 was localized in prostate cancer stroma, and no immunostaining was seen in prostatic epithelial cells. Giri et al. (42) documented that FGF2 was present in the stromal fibroblasts and endothelial cells but not the prostate cancer cells. These findings further supported the notion that FGF2 in prostate cancer tissue was derived from stromal cells rather than prostate cancer cells.

In this study, we demonstrated that co-culture with HUVEC did not increase FGF2 expression in 22Rv1 and C4-2B cells in the co-culture system within 48 h. Moreover, FGF2 was mainly derived from HUVEC cells in the co-culture system. These results were in agreement with Muir and Russo’s studies that FGF2 could hardly be detected in 22Rv1 and C4-2B cells (43, 44). Endothelial cells as the main source of FGF2 are consistent between our *in vitro* co-culture model and *in vivo* studies from Sinowatz and Giri (41, 42). However, in view of the limited co-culture time and capacity, we can not exclude the possibility that HUVEC will precondition prostate cancer cells to produce FGF2 in the co-culture system with prolonged co-culture.

Furthermore, we confirmed that FGF2 expression in HUVEC and prostate cancer cells in the co-culture system did not rise following the indicated docetaxel treatment, which indicated that the crosstalk between prostate cancer cells and HUVEC cells was the major factor that determined the secretion of FGF2. However, due to the limited exposure dose and time to docetaxel, we can not rule out the possibility that docetaxel will contribute to FGF2 production in the co-culture system with increased dosages or prolonged incubation.

In addition, we demonstrated that FGF-R expression in prostate cancer cells did not augment whether co-cultured with HUVEC or following docetaxel treatment, which suggested that the increasing FGF2 derived from HUVEC cells was the primary factor that contributed to the FGF2/FGFR signaling pathway. However, given the limited exposure dose and time to docetaxel or co-culture duration, we can not preclude the possibility that HUVEC or docetaxel contributes to FGFR production in prostate cancer cells with increased dosages or prolonged incubation. Taken together, these findings indicated the significance of FGF/FGFR signaling through paracrine FGF2 in a cancer microenvironment for the development and progression of prostate cancer. However, we can not obviate the possibility that FGF2 secreted by prostate cancer cells may occur in more advanced prostate cancer because most of the immunohistochemical studies of FGF2 focused on radical prostatectomy specimens. Our future approach to determining whether cancer-derived FGF2 is critical in tumor progression is to use knockout mice with either prostate-specific or whole-mouse deletion of FGF2 to determine whether prostate cancer progression in transgenic mouse models is affected.

FGF2 has been manifested to activate Akt/mTOR signaling pathway in earlier studies (45, 46). In this study, we confirmed that ERG was able to induce Akt phosphorylation, which was consistent with the previous report (47). The activation of the Akt/mTOR pathway contributed to the docetaxel resistance of prostate cancer, which was corroborated in previous studies (48, 49). Herein, docetaxel resistance of ERG-overexpressing prostate cancer cells was significantly reversed when Perifosine, the inhibitor of the Akt/mTOR signaling pathway was added, which was in agreement with the previous report (32). Our recent findings and previous reports will pave the way for further study of Akt inhibitors combined with docetaxel in the treatment of CRPC.

However, the mouse xenograft tumors we obtained were generally ERG negative by immunohistochemical analysis. There might be two reasons to account for this result. First, the mouse xenograft tumors were primarily composed of 22Rv1 cells which were generally ERG negative without any treatment (50, 51). We determined ERG expression in 22Rv1 cells co-cultured with HUVEC by Western blotting *in vitro*. Nevertheless, ERG expression in 22Rv1 cells might not be elevated to strong enough to be identified by immunohistochemical analysis *in vivo* because the latter may not be as sensitive as Western blotting to detect protein expression (52). Second, ERG expression is under complex regulation *in vivo*. ERG expression in prostate cancer is variable and is associated with activation of multiple pathways and proteins including the MYC, NFkB, AR pathways and SRC1, Sprouty1, SKP2  (53, 54). Some other factors such as DNA damage may also affect the expression of ERG *in vivo* besides FGF2 (55). Although there are some findings in this study, there are several limitations as well. First, we could not investigate the FGF2-ERG-Akt/mTOR signaling axis in clinical samples due to the availability of CRPC specimens. Second, ERG expression status could not be verified *in vivo* because the mouse xenograft tumors we obtained were generally negative in immunohistochemical staining for ERG. Third, we did not acquire the direct molecular evidence to show how FGF2 activates ERG expression in detail. Fourth, we did not validate the efficacy of FGF2 antagonists to overcome docetaxel resistance *in vivo* due to limited funding. Notwithstanding these inadequacies, we still demonstrate that endothelial cells-derived FGF2 plays an important part in promoting docetaxel resistance of prostate cancer cells through *in vitro* and *in vivo* experiments.

In summary, our findings highlight that endothelial cells play a significant role in enhancing the proliferation and inhibiting the docetaxel-induced apoptosis of prostate cancer cells *in vitro* and *in vivo*. Specifically, we demonstrated that FGF2 secreted from endothelial cells can upregulate ERG expression in prostate cancer, which then activates the Akt/mTOR signaling pathway and promote docetaxel resistance of prostate cancer subsequently. Besides, we propose that endothelial cells, as a key component of the tumor microenvironment, contribute to docetaxel resistance of prostate cancer cells in a paracrine fashion, which may provide new insights for the development of novel anticancer therapies. Furthermore, evidence from *in vitro* studies suggests that inhibitors of FGF/FGFR signaling combined with docetaxel warrant further investigation as a potential therapeutic option for the treatment of patients with advanced prostate cancer.

## Data Availability Statement

The original contributions presented in the study are included in the article/**Supplementary Material**; further inquiries can be directed to the corresponding authors.

## Ethics Statement

The animal study was reviewed and approved by the Medical Science Ethics Committee of Shanghai General Hospital.

## Author Contributions

BH, HL, and XW conceptualized and designed the study. WZ and XW developed the methodology. WZ, YS, and YZ acquired the data. WZ, YS, and YZ analyzed and interpreted the data. BH, HL, and XW supervised the study, WZ and YS prepared the manuscript. All authors contributed to the article and approved the submitted version.

## Funding

This work was supported by the Natural Science Foundation of Shanghai, China (Grant NO:19ZR1441200).

## Conflict of Interest

The authors declare that the research was conducted in the absence of any commercial or financial relationships that could be construed as a potential conflict of interest.

## Supplementary Material

Supplementary Figure 1 Expression of FGF2 in prostate cancer cells and HUVEC cells cultured alone or with each other. **(A)** Quantitative PCR analysis of FGF2 expression in 22Rv1 cultured with or without HUVEC cells for 48h. **(B)** Quantitative PCR analysis of FGF2 expression in C4-2B cultured with or without HUVEC cells for 48h. **(C)** Quantitative PCR analysis of FGF2 expression in 22Rv1 and HUVEC cells cultured with each other for 48h. **(D)** Quantitative PCR analysis of FGF2 expression in C4-2B and HUVEC cells cultured with each other for 48h.

Supplementary Figure 2 Expression of FGF2 in HUVEC cells and prostate cancer cells cultured with each other with or without docetaxel treatment (10nM for 48h). **(A)** Quantitative PCR analysis of FGF2 expression in HUVEC cells cultured with 22Rv1 with or without docetaxel treatment (10nM for 48h). **(B)** Quantitative PCR analysis of FGF2 expression in HUVEC cells cultured with C4-2B with or without docetaxel treatment (10nM for 48h). **(C)** Quantitative PCR analysis of FGF2 expression in 22Rv1 cells cultured with HUVEC with or without docetaxel treatment (10nM for 48h). **(D)** Quantitative PCR analysis of FGF2 expression in C4-2B cells cultured with HUVEC with or without docetaxel treatment (10nM for 48h).

Supplementary Figure 3 Expression of FGFR in prostate cancer cells cultured with or without HUVEC cells with or without docetaxel treatment (10nM for 48h). **(A)** Quantitative PCR analysis of FGFR expression in 22Rv1 cultured with or without HUVEC cells for 48h. **(B)** Quantitative PCR analysis of FGFR expression in C4-2B cultured with or without HUVEC cells for 48h. **(C)** Quantitative PCR analysis of FGFR expression in 22Rv1 cultured with HUVEC cells with or without docetaxel treatment (10nM for 48h). **(D)** Quantitative PCR analysis of FGFR expression in C4-2B cultured with HUVEC cells with or without docetaxel treatment (10nM for 48h).
