# Supplementary figures and images for: Endothelial Cells Promote Docetaxel Resistance of Prostate Cancer Cells by Inducing ERG Expression and Activating Akt/mTOR Signaling Pathway
Source: Front Oncol. 2020 Dec 16;10:584505. doi: 10.3389/fonc.2020.584505 (PMC7793734; doi:10.3389/fonc.2020.584505)

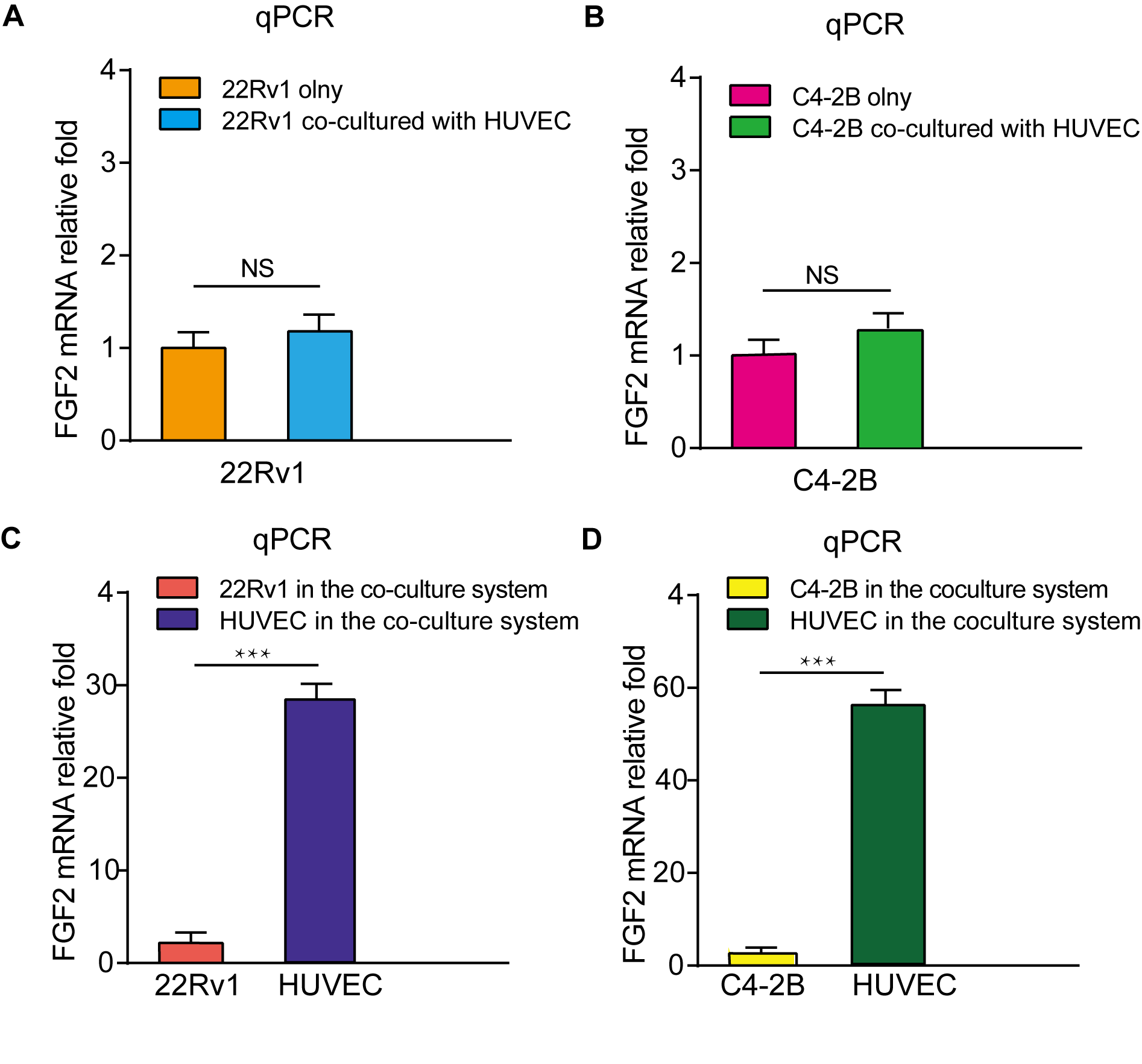

Supplement: Supplementary Figure 1 — Expression of FGF2 in prostate cancer cells and HUVEC cells cultured alone or with each other. (A) Quantitative PCR analysis of FGF2 expression in 22Rv1 cultured with or without HUVEC cells for 48h. (B) Quantitative PCR analysis of FGF2 expression in C4-2B cultured with or without HUVEC cells for 48h. (C) Quantitative PCR analysis of FGF2 expression in 22Rv1 and HUVEC cells cultured with each other for 48h. (D) Quantitative PCR analysis of FGF2 expression in C4-2B and HUVEC cells cultured with each other for 48h. [file Image_1.tif]

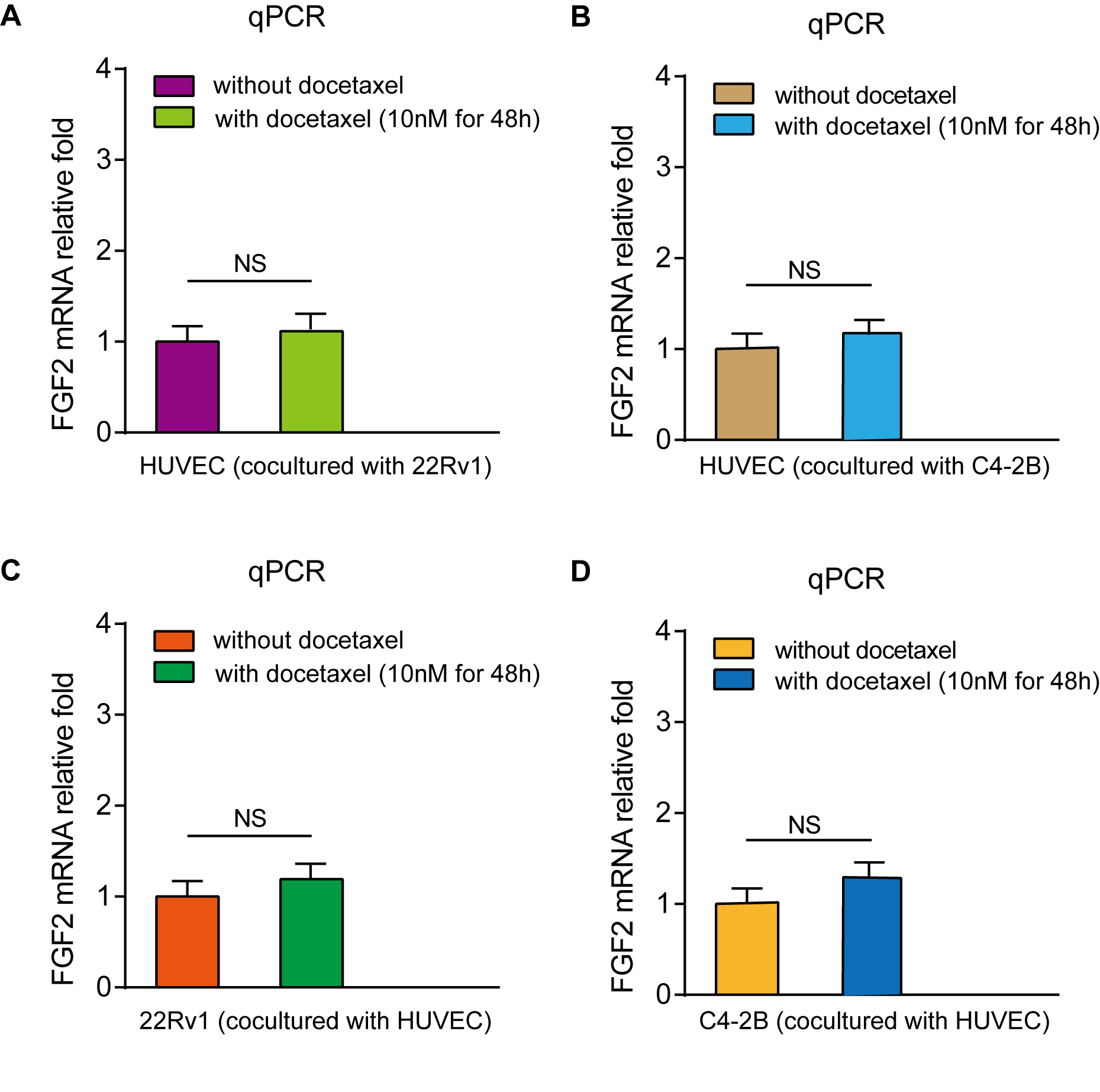

Supplement: Supplementary Figure 2 — Expression of FGF2 in HUVEC cells and prostate cancer cells cultured with each other with or without docetaxel treatment (10nM for 48h). (A) Quantitative PCR analysis of FGF2 expression in HUVEC cells cultured with 22Rv1 with or without docetaxel treatment (10nM for 48h). (B) Quantitative PCR analysis of FGF2 expression in HUVEC cells cultured with C4-2B with or without docetaxel treatment (10nM for 48h). (C) Quantitative PCR analysis of FGF2 expression in 22Rv1 cells cultured with HUVEC with or without docetaxel treatment (10nM for 48h). (D) Quantitative PCR analysis of FGF2 expression in C4-2B cells cultured with HUVEC with or without docetaxel treatment (10nM for 48h). [file Image_2.tif]

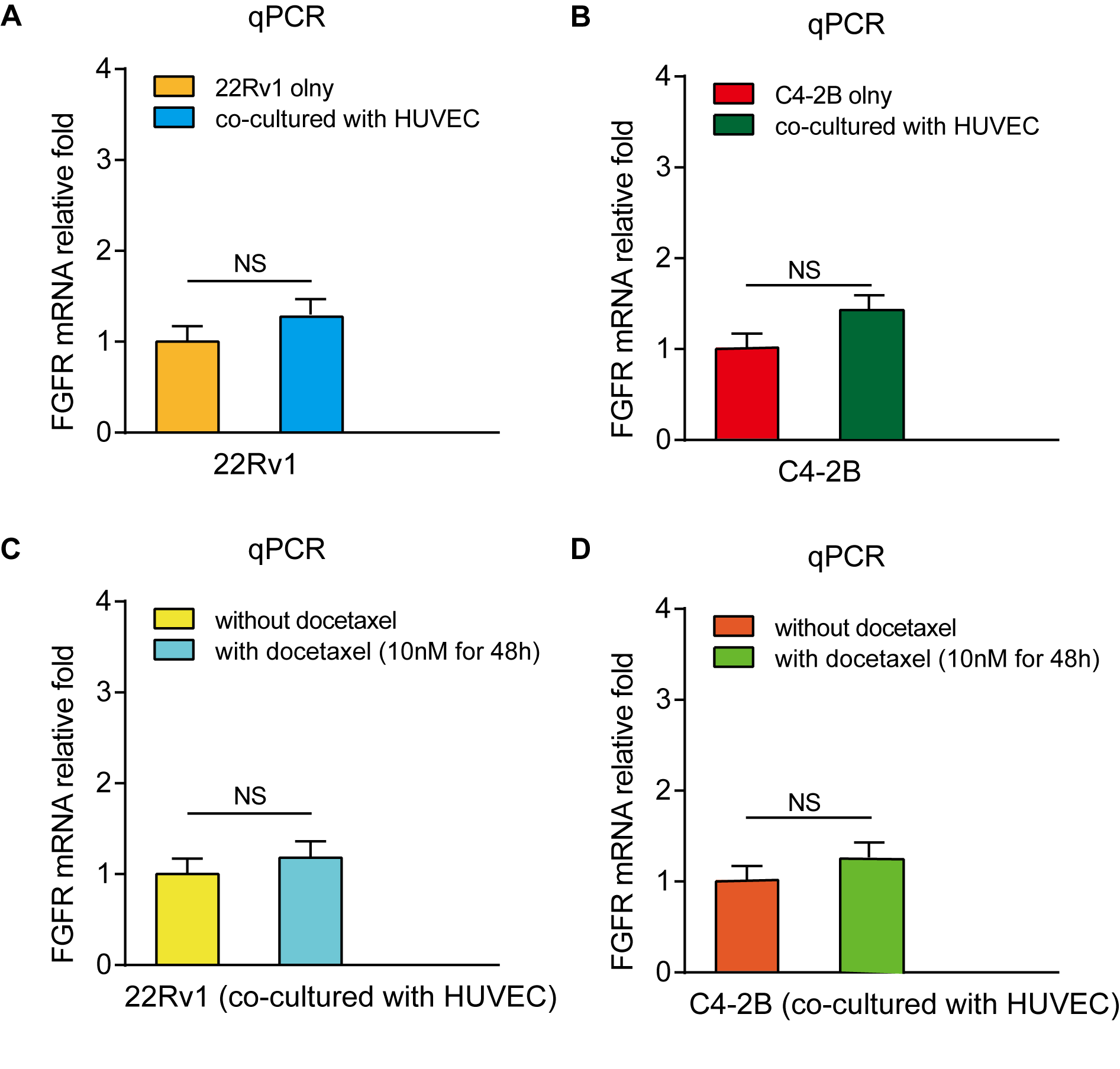

Supplement: Supplementary Figure 3 — Expression of FGFR in prostate cancer cells cultured with or without HUVEC cells with or without docetaxel treatment (10nM for 48h). (A) Quantitative PCR analysis of FGFR expression in 22Rv1 cultured with or without HUVEC cells for 48h. (B) Quantitative PCR analysis of FGFR expression in C4-2B cultured with or without HUVEC cells for 48h. (C) Quantitative PCR analysis of FGFR expression in 22Rv1 cultured with HUVEC cells with or without docetaxel treatment (10nM for 48h). (D) Quantitative PCR analysis of FGFR expression in C4-2B cultured with HUVEC cells with or without docetaxel treatment (10nM for 48h). [file Image_3.tif]
